# The Relationship between Cancer Caregiver Burden and Psychological Outcomes: The Moderating Role of Social Connectedness

**DOI:** 10.3390/curroncol29010002

**Published:** 2021-12-22

**Authors:** Eva Y. N. Yuen, Carlene J. Wilson

**Affiliations:** 1 Institute for Health Transformation, Deakin University, Burwood, VIC 3125, Australia; 2 Centre for Quality and Patient Safety, Monash Health Partnership, Monash Health, Clayton, VIC 3168, Australia; 3 Olivia Newton-John Cancer, Research and Wellness Centre, Psycho-Oncology Research Institute, Austin Health, Heidelberg, VIC 3084, Australia; c.wilson2@latrobe.edu.au; 4 School of Psychology and Public Health, La Trobe University, Bundoora, VIC 3086, Australia; 5 College of Education, Psychology and Social Work, Flinders University, Bedford Park, SA 5042, Australia

**Keywords:** caregivers, cancer, social connectedness, depression, anxiety, moderation analysis, wellbeing

## Abstract

The present study: (a) examined the extent of caregiver burden and psychological wellbeing and (b) tested whether social connectedness moderated the association between caregiver burden and psychological symptoms in caregivers of people with cancer. The cross-sectional survey study included 189 cancer caregivers (mean age = 36.19 years, standard deviation = 11.78; 80.4% female). Data were collected on caregiver burden, social connectedness, and depression and anxiety. Moderation analysis was conducted to examine the effect of social connectedness on the relationship between caregiver burden and depression and anxiety. Caregiver burden was positively associated with depression and anxiety symptoms. Controlling for significant demographic and caregiver characteristics, the moderation model showed as perceived social connectedness increased, the relationship between caregiver burden and depression decreased (β = −0.007, se = 0.004, 95% CI: −0.014, 0.000, *p* = 0.05). By contrast, social connectedness did not moderate the association between caregiver burden and anxiety. Findings have implications for the management of depression in cancer caregivers. Social connectedness appears to provide a protective buffer from the negative impacts of caregiving, providing increased psychological resources to manage the burden associated with caregiving, resulting in lower depression. Research on strategies to improve caregiver wellbeing through enhancing engagement with social networks in ways that improve perceived sense of connectedness with others is warranted.

## 1. Introduction

Family members and friends often provide vital care that includes practical, physical, psychosocial and financial support to people living with cancer [1,2]. With provision of supportive care increasingly shifting from the formal health system to the home, these individuals, often referred to as informal caregivers in the research literature, are increasingly expected to undertake care traditionally delivered by trained providers [3,4]. These often sudden and complex caregiving responsibilities can adversely impact caregivers’ own health and wellbeing. Indeed, research has shown that caregivers are often under considerable strain, resulting in threats to psychological and physical wellbeing [5,6]. 

Although positive outcomes following caregiving have been reported, including an improved relationship with the care recipient, providing care for someone with cancer has simultaneously been described as stressful, demanding, and burdensome [6,7]. Caregiver burden is typically described as perceived emotional, social, physical, financial, and/or spiritual strain as a result of providing care [8]. A recent review confirms the association between high caregiver burden and poor psychological outcomes in cancer populations of all ages [9]. The review highlighted that perceived caregiver burden was associated with negative impacts on work productivity and finances, changes in social and family relationships, and increased needs for direct intervention and support [9]. Caregiver burden can also impact the capacity of caregivers to provide quality care and consequent health outcomes for care recipients. In a recent cross-sectional study of caregiver-patient dyads, higher caregiver burden was associated with preference for palliative care over life sustaining treatment for the care recipient [10]. In a longitudinal study of community-dwelling caregiver-care recipient dyads, high caregiver burden was associated with greater likelihood of mortality and hospitalization in dependent adults at the 3-year follow-up [11]. Identifying potentially modifiable factors that reduce caregiver burden and improve caregiver psychological outcomes has the potential to improve caregiver health and wellbeing, as well as to improve health outcomes for people with cancer. 

Across community and health populations, social connectedness has been shown to be an important factor in promoting optimal psychological wellbeing. Although the concepts social connectedness and social support are interrelated, there are theoretical differences. Social connectedness is a perception or feeling held by a person and has been described as the opposite of loneliness [12,13,14]. It reflects an individual’s subjective sense of meaningful, close, and constructive interpersonal relationships with other individuals, groups or society, as reflected in: caring about others, and feeling cared about by others (e.g., love, companionship, or affection), and, feelings of belongingness to a group or community [12,13,14]. By contrast, social support is understood as a multidimensional construct that includes provision of different types of support, including: emotional, instrumental, appraisal, and informational [14]. Lee and Robbins [15] argued that social connectedness differs from social support because the former captures a persistent and pervasive worldview of how an individual relates to their social environment, rather than the quality or quantity of specific types of relationships; thus, even individuals who identify as having long-term relationships and friendship networks may still report feeling a lack of connectedness, or belongingness in their lives. 

Low social connectedness has been associated with greater risk of depression in older adults [16], whereas higher perceived social connectedness has been positively associated with subjective wellbeing [17], and self-esteem and wellbeing in adolescents [18]. Social connectedness has also been posited to buffer the negative effects of stressful or traumatic life events. Recent studies found that social connectedness moderated the relationships between war-related post-traumatic stress disorder and poorer health [19], and poor cognitive functioning [20] in older adults, suggesting the protective role of social connectedness in the context of exposure to traumatic events.

Although studies have shown that lack of perceived social support is positively associated with depression and anxiety in caregivers [21], currently little research examines whether perceived social connectedness impacts psychological health outcomes in caregivers and, if so, how. Further, although the relationship between caregiver burden and psychological symptoms is well established [9], there exists a gap in understanding whether social connectedness interacts to moderate this relationship. An understanding of factors that moderate the relationship between caregiver burden and psychological wellbeing would enable researchers to develop effective interventions to assist caregivers in reducing depression and anxiety symptoms as they provide care to a family member or friend. 

The aim of the current study was to assess the extent of caregiver burden and psychosocial outcomes in a sample of cancer caregivers, and to examine the potential moderating effect of perceived social connectedness on this relationship. The study hypotheses were: (a) caregiver burden is positively associated with depression and anxiety symptoms, and (b), perceived social connectedness moderates the relationship, such that greater perceived social connectedness is associated with a smaller association between caregiver burden on psychological morbidity. 

## 2. Materials and Methods

### 2.1. Participant Recruitment

An online survey developed and delivered via RedCap was approved by the (redacted) Human Ethics Committee (Approval # HEC20295). Participants were recruited through Prolific (www.prolific.co; accessed 1 October 2020), a crowdsourcing research platform developed in 2014 primarily targeting researchers and startup companies, which is supported by Oxford University Innovation [22]. Prolific statistics from October 2021 showed that 211,929 individuals from the UK, US, Australia, New Zealand, and Canada were active on the Prolific platform in the past 90 days. To generate participant samples on Prolific, researchers have the option to: (a) filter participants using demographic and/or health related screeners (e.g., sex, age, chronic disease type), (b) create custom screeners, or (c) request a representative sample of the US or UK (www.prolific.co; accessed 1 October 2020). Participants are reimbursed for their time, with the platform recommending £5.00 to £7.50 per hour. In a recent study that compared three crowdsourcing platforms (Prolific, MTurk, and Crowdflower) with a university sample, Peer and colleagues [22] found that across a range of tasks and experiments, Prolific and MTurk replicated existing findings and provided higher data quality compared to Crowdflower and the university sample. Further, Peer and colleagues [22] found Prolific participants were less dishonest, more naïve to research tasks, and were more geographically and ethnically diverse compared to the two other crowdsourcing platforms. Thus, for the current study, caregivers were recruited through Prolific between October-November 2020. 

To recruit participants from Prolific, a two-step process was conducted to identify a sample of caregivers of people with cancer. Firstly, a pre-screening survey was administered to identify people who: (a) had provided care to an adult with cancer in the preceding 5 years, (b) were both aged 18+ years, and (c) were residing in the UK, Canada, USA, Australia, or New Zealand. Individuals who met the inclusion criteria were re-invited to participate in the main study through Prolific using their non-personally identifiable Prolific ID. Participants provided informed consent at both stages of the recruitment process and received $0.18AUD at the completion of the pre-screening survey, regardless of whether they met the study criteria. Those who were re-invited for the main study received the equivalent of $10AUD following survey completion. 

### 2.2. Measures

#### 2.2.1. Sociodemographic Characteristics

Data collected on caregiver sociodemographics included age, gender, and educational attainment. Illness and disability were assessed as the number of self-reported health-related conditions. 

#### 2.2.2. Care-Related and Care Recipient Characteristics

Care-related information collected included relationship to the care recipient, length of time providing care, and resident vs. non-resident caregiving status (e.g., “Do you currently live with your family member or friend with cancer?”). Care recipient information collected included cancer diagnosis type, time since diagnosis, and whether they were currently receiving treatment for cancer.

#### 2.2.3. Caregiver Burden

Caregiver burden (predictor variable) was assessed using the short version of the Burden Scale for Family Caregivers (BSFC-s) [23]. The 10-item short form scale was designed to measure subjective burden in informal caregivers, using a 4-point Likert scale (strongly disagree-strongly agree). The total score ranges from 0 to 30, with higher scores indicating greater burden. Cronbach alpha for the BSFC-s in the current study was 0.89. 

#### 2.2.4. Social Connectedness 

Social connectedness (moderator variable) was assessed using the Social Connectedness Scale-Revised [24]. The 8-item scale examines individual perceptions of feeling connected with others and society on a 6-point Likert scale (strongly disagree–strongly agree), with higher scores indicating greater perceived connectedness. For the current study, a 5-point scale with a midpoint (neither agree nor disagree) was used to reduce the response options. Internal consistency of the scale in the current study was adequate (Cronbach’s alpha = 0.94). 

#### 2.2.5. Depression and Anxiety 

Depression and anxiety symptoms (outcome variables) were assessed using the 14-item Hospital Anxiety and Depression Scale (HADS) [25]. Studies have shown the utility of the scale across general populations [26,27,28]. The scale is comprised of two sub-scales, each of seven items that measure depression and anxiety on a 4-point scale (0–3). Subscale scores are summed across the seven items (range = 0–21), with higher scores indicating greater anxiety and depression. For the current study, scores ≥8 were used to define greater likelihood of the presence of depression and anxiety symptoms [29]. Cronbach alpha for the depression and anxiety subscales in the current study were 0.66 and 0.83, respectively. 

#### 2.2.6. Statistical Analysis

All statistical analyses were performed using SPSS v26. Complete data were available for 189 participants. Means were calculated for continuous variables and frequencies for categorical variables. The relationship of the care recipient to caregiver was coded into three categories for the analyses: spouse, other family member, or friend. Residency status of caregivers was dichotomized (yes, no). Marital status was dichotomized (in a relationship; not in a relationship). Educational attainment was dichotomized for analyses (High school or less, and vocational education; undergraduate and postgraduate education). 

Associations between the main study variables (caregiver burden, social connectedness, depression, anxiety) and sociodemographic (age, gender, educational attainment, illness/disability) and care-related variables (lives with care recipient, caregiving duration, relationship to the care recipient, care recipient currently receiving treatment) were initially examined using Pearson correlation, independent samples *t*-test and chi-square analyses. Sociodemographic and care-related variables identified as significantly correlated with the main study variables (*p* ≤ 0.05) were included in subsequent analyses as control variables. 

Moderation analysis was conducted using the PROCESS macro for SPSS (version 3.3) [30], to investigate whether perceived social connectedness had moderating effects on the relationship between caregiver burden and depression and anxiety symptoms, controlling for sociodemographic and care-related variables identified as significantly associated with the outcome variables in the bivariate analyses. When significant moderation effects were identified, linear interactions were plotted with simple slopes at low, medium, and high levels of the moderator (16th, 50th, and 84th percentiles, respectively). The Johnson–Neyman technique [31] was also used to determine the specific value of the moderator variable (social connectedness) where the interaction was statistically significant. 

## 3. Results

### 3.1. Participants

A total of 1010 individuals completed the pre-screening survey; 300 met the inclusion criteria (i.e., identified as providing care to someone with cancer in the preceding 5 years, both aged 18+), and were re-invited to complete the study survey. Of the participants who commenced the survey (*n* = 234), 20 cases who made no progress following the consent page were removed. An additional 25 participants who did not complete items for the main study variables were also excluded. Table 1 shows the sociodemographic details of the remaining 189 participants. Over one-third of participants were resident caregivers (34.4%; *n* = 65). The mean age for all participants was 36.19 years (SD = 11.78), and over four-fifths were female. The mean age for resident caregivers was 36.19 years (SD = 11.78; range: 18–74), with over two-thirds female (see Table 1). Mean time since diagnosis for care recipients as reported by the caregiver was 2.76 years (SD = 3.94), while the mean duration of caregiving was 2.44 years (SD = 3.37). Most participants spoke English at home (99.5%).

### 3.2. Preliminary Data Analyses

#### 3.2.1. Relationships between Caregiver Sociodemographic and Care-Related Characteristics 

The relationship between caregiver sociodemographic variables (age, gender, number of caregiver illnesses/disabilites) and care-related characteristics (relationship to the care recipient, living with or separate from the care recipient, whether the care recipient was receiving treatment, and duration of caregiving) were explored. Age was not associated with relationship to the care recipient (F[2, 186] = 0.149, *p* = 0.861), living circumstances (t[187] = 1.43, *p* = 0.153), treatment status of the care recipient treatment (t[187] = 0.60; *p* = 0.546) nor the duration of caregiving (r[186] = 0.124; *p* = 0.09).

Chi square analyses showed no differences between genders for relationship with the care recipient (X^2^ = [2, 188] = 0.193, *p* > 0.05), however women were more likely than men to live with the care recipient (X^2^ = [1, 188] = 4.68, *p* = 0.03). Women were also more likely to be caring for someone currently receiving treatment (X^2^ = [1, 188] = 4.72, *p* = 0.03). Gender was not associated with duration of caregiving (t[185] = 1.31; *p* = 0.191).

When comparing the association between extent of the caregivers ill health and disability and the circumstances of care, no differences were linked to the caregiver-care-recipient relationship (F[2, 186] = 1.28; *p* = 0.281), place of residence of caregiver (with or away from care recipient; t[187] = 0.23; *p* = 0.819), or treatment completion status (i.e., in treatment or completed treatment; t[187] = 0.28; *p* = 0.777). Extent of caregiver illness or disability was not correlated with duration of caregiving (r[186] = 0.12; *p* = 0.102).

#### 3.2.2. Relationships between Main Study Variables (Depression, Anxiety, Burden, Social Connectedness) and Sociodemographic Characteristics

Table 2 shows the means, standard deviations, and correlations for the main study variables, including correlations with demographic and care-related characteristics. Examination of associations between caregiver burden, social connectedness, depression, anxiety, and sociodemographic variables found that age, gender, and caregiver illness/disability were significantly associated with depression and anxiety. Age was negatively associated with anxiety (r[187] = −0.155, *p* = 0.03); however, it was not significantly correlated with depression, caregiver burden, or social connectedness. Women were significantly more likely to report higher levels of anxiety (*n* = 152; M = 11.77, SD = 3.87; t[186] = −2.61, *p* = 0.01) than men (*n* = 36; M = 9.86, SD = 4.28). No differences were found between genders for depression, caregiver burden, or social connectedness. Caregiver illness/disability was positively and significantly correlated with depression (r[187] = −0.19, *p* < 01) and anxiety (r[187] = −0.16, *p* = 0.03), however it was not significantly associated with caregiver burden or social connectedness. No significant associations were found between educational attainment and the main study variables. As such, age, gender, and caregiver illness disability were included as control variables in the moderation analysis with anxiety as the outcome variable; only caregiver illness/disability was included as a control variable with depression as the outcome variable.

#### 3.2.3. Relationships between Main Study Variables (Depression, Anxiety, Social Connectedness, Burden) and Care-Related Characteristics

Significant associations were found between the main study variables and care-related factors, specifically, the relationship to the care recipient, if they live with the care recipient, and whether the care recipient was currently receiving treatment.

The relationship between caregiver and recipient (spouse; family member; friend) influenced caregivers’ experience of caring, with statistically significant differences for depression (F[2, 186 = 4.85], *p* < 0.01; effect size [ES] = 0.05), caregiver burden (F[2, 186] = 4.24, *p* < 0.02; ES = 0.04) and social connectedness (F[2, 186 = 3.13], *p* < 0.05; ES = 0.03). No significant associations were observed for anxiety. For depression, Tukey post-hoc comparisons revealed that spousal caregivers reported significantly higher levels of depression (M = 11.0, SD = 3.61) compared to family (M = 8.91, SD = 3.08, *p* = 0.02) and non-family caregivers (M = 8.31, SD = 3.08, *p* < 0.01). No significant difference was found between family and non-family caregivers for depression (*p* = 0.56). For caregiver burden, spousal caregivers reported significantly higher burden (M = 18.7, SD = 5.60) compared to family (M = 15.12, SD = 5.31, *p* = 0.02) and non-family (M = 14.92, SD = 5.35, *p* = 0.03) caregivers. No significant difference was found between family and non-family caregivers (*p* = 0.988) in caregiver burden. For social connectedness, post-hoc comparisons found that spousal caregivers reported lower social connectedness (M = 22.1, SD = 6.20) compared to non-family (M = 26.95, SD = 8.47; *p* = 0.05), with the difference between spousal and family (M = 26.37, SD = 7.45; *p* = 0.052) caregivers approaching significance.

Caregivers who lived with the care recipient reported significantly more depression, anxiety, caregiver burden, and less social connectedness compared to those who did not live with the care recipient (See Table 3). A chi-square test revealed a significant relationship between residential status and relationship with the care recipient (X^2^[2, *n* = 189] = 45.13, *p* < 0.001). Given the likelihood of spousal caregivers also residing with the care recipient, and residency status being significantly associated with all main study variables, only residency status (not relationship with the care recipient) was included as a covariate in the moderation analyses.

Caregivers providing care to someone currently receiving treatment were more likely to report higher social connectedness (*n* = 155; M = 25.52, SD = 7.61; t[187] = 2.03, *p* = 0.04], compared to caregivers providing care to someone not currently receiving treatment (*n* = 34; M = 28.44, SD = 7.53). Care recipient treatment status was not associated with caregiver depression, anxiety, or burden. No significant associations were found for ‘caregiving duration’ or ‘time since care recipient was diagnosed’ and the main study variables.

Thus, resident status and care recipient currently receiving treatment were included as control variables in the moderation analyses with depression or anxiety as the outcome variable.

#### 3.2.4. The Moderating Effect of Social Connectedness on the Relationship between Caregiver Burden and Depression and Anxiety Symptoms

**Figure 1:**
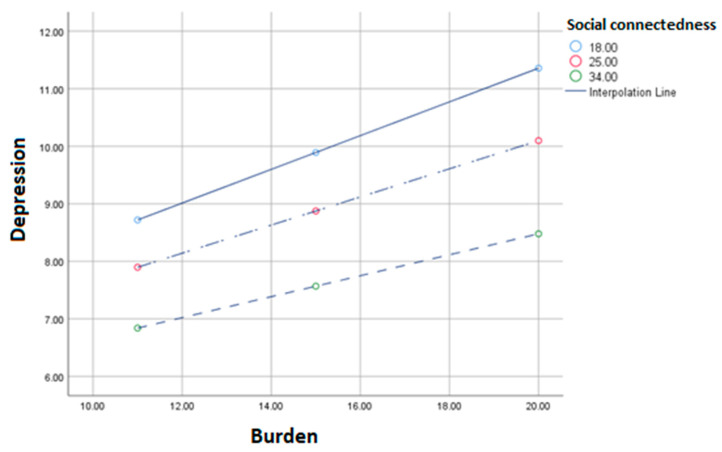
Moderation of the relationship between caregiver burden and depression symptoms when perceived social connectedness is low (18), medium (25), or high (34).

The moderation model with depression as the outcome variable showed that after controlling for significant demographic and caregiver characteristics (caregiver illness/disability, resident status, care recipient in active treatment), as perceived social connectedness increases, the relationship between caregiver burden and depression decreases (β = −0.007, se = 0.004, 95% CI: −0.014, 0.000, *p* = 0.04; See Table 4, Figure 1). That is, for every one unit increase in social connectedness, there was a decrease in the slope between burden and depression of 0.007. The Johnson-Neyman technique revealed no significant transition points, suggesting that the moderating effect of social connectedness for all scores (8–40; 100% of the sample) was not restricted to a specific range of caregiver burden. By contrast, the moderation analysis with anxiety as the outcome variable showed that after controlling for significant demographic and caregiver characteristics (age, gender, caregiver illness/disability, residential status, care recipient in active treatment) social connectedness did not significantly moderate the association between caregiver burden and anxiety symptoms (β = −0.003, se = 0.004, 95% CI: −0.012, 0.005, *p* = 0.46).

## 4. Discussion

The current study aimed to examine the relationship between caregiver burden and psychological symptoms in caregivers of people with cancer, and to test the moderating effect of perceived social connectedness on this relationship. The study hypotheses were partially supported: (a) caregiver burden was positively associated with depression and anxiety symptoms, and (b) after controlling for significant demographic and care-related characteristics, perceived social connectedness significantly moderated the relationship between caregiver burden and depression, such that the effect of burden on depression symptoms was smaller with higher levels of perceived social connectedness. By contrast, perceived social connectedness did not significantly moderate the relationship between caregiver burden and anxiety.

Findings from the study support existing literature and is the first step in advancing our understanding of the impacts of social connectedness on caregiver wellbeing in several ways. While social support has been identified as being associated with psychological outcomes in caregivers [21,32], our finding that social connectedness buffers the relationship between caregiver burden and depression symptoms is novel. The results suggest that caregivers who perceive higher social connection were at least partially protected from the negative impacts of caregiver burden. This suggests that higher perceived connectedness with others was likely to provide caregivers with increased psychological resources to manage the burden associated with caregiving, resulting in lower depression symptoms. Our findings are consistent with previous studies [32,33] that described social connectedness as a protective buffer between challenging circumstances and depression [34]. In a recent pilot study, Taylor and colleagues [35] found that a positive activity intervention program (comprised of exercises derived from positive psychology designed to promote positive thoughts, behaviors, and emotions) was effective in enhancing social connectedness, improving positive emotions, and decreasing negative emotions in a small sample (*n* = 29) of people with clinically impairing depression or anxiety. Further research on whether similar programs implemented within caregiver populations could minimize symptoms of depression through improved social connectedness is warranted.

The unexpected finding that social connectedness did not moderate the relationship between caregiver burden and anxiety may be explained by differences in the mechanisms by which social support influences anxiety. For example, in a recent longitudinal study, Garcia-Torres and colleagues [32] found that some domains of social support, specifically, levels of support-seeking and lack of informational support, predicted anxiety in caregivers within the first 6 months of diagnosis. It is possible that social connectedness (i.e., a sense of belongingness and meaningful relationships) may not alleviate anxiety related to uncertainty, fears, and worries in response to managing a cancer diagnosis, which may be better controlled through access to adequate information. Further research into types of social support, and whether perceived access to adequate information buffers the impacts of burden on caregiver anxiety is warranted.

In support of existing research [36,37], the current study revealed that spousal caregivers, and those who lived with the care recipient reported higher caregiver burden and more depression and anxiety symptoms. It is likely that these caregivers are required to provide more support with activities of daily living, to manage caregiving alongside family and work responsibilities [38], and had little choice in undertaking the caregiving role. Our findings suggest that spousal and co-resident caregivers may require additional supports to effectively manage the demands of their caregiving role.

Our findings also showed that different caregiver characteristics were associated with different self-reported levels of social connectedness. Spousal caregivers and those who lived with the care recipient reported lower perceived social connectedness. Lack of time due to caregiving responsibilities, perceived inability to feel understood as a caregiver, changes in social networks, as well as receipt of practical but not emotional support, have been identified through qualitative research as negative social consequences of providing care [39,40]. These consequences may all result in caregivers perceiving lower social connectedness with others. Interestingly, caregivers providing care to someone in active treatment were more likely to report higher social connectedness, compared to those whose care recipient was not receiving treatment. This finding is consistent with a recent study of older adults with depression, that reported increased social connectedness in respondents who were caregivers compared to those who were not [41]. There is the possibility that caregivers in the current study perceived increased closeness with their care recipient, and/or increased their interaction with supportive health providers and community when their care recipient was in active treatment, which increased their perception of connectedness with others. Alternatively, Given and colleagues [42] posited that during active treatment, caregivers may focus their activities and attention on providing care, foregoing social and personal activities; once treatment has ended, caregivers may experience difficulties reintegrating into their formal social life and restoring social connections as the relationships, social support networks and opportunities may no longer exist. Thus, caregivers in the current study may have experienced a heightened loss of social connection after their care recipient’s completed treatment. Findings suggest caregivers may also need support with transitioning following their care recipient’s treatment completion, particularly when the caregiver is a spouse.

### Limitations of the Study

The current study has several limitations. The data were collected through a crowdsourcing research platform, which could limit the generalizability of the findings to caregivers with computer and internet proficiency, and who register on such panels. Indeed, respondents were predominantly younger females, highly educated, and spoke English at home. Further, a large proportion of caregivers were adult children providing care to their parent recently diagnosed with cancer and still in treatment, which differs from other studies of caregiver burden, which have tended to recruit older, spousal samples [43,44]. Consequently, the currently described experiences of caregiving are likely to have differed significantly from caregivers who were older, a spousal caregiver, had less education, or were from culturally and linguistically diverse backgrounds. There is the potential that older spousal caregivers, and those whose care recipient was no longer receiving treatment, may have fewer support networks and less social connectedness. Consequently, this sample may be at increased risk for anxiety and depression associated with caring. We did not collect data on cancer stage, nor family income or ethnicity, which are all important determinants of health. Moreover, given that the sample was derived from the community, likely with a range of health service experiences, different findings may be evident from participants recruited from specific clinical settings. In addition, the survey data were collected during the COVID-19 pandemic, which may have impacted caregivers’ capacity to make meaningful connections within their social networks, and thus, impacted their scores on perceived social connectedness. Finally, the study was cross-sectional, and as such causal effects cannot be inferred. Longitudinal studies are needed to understand the causal impacts of social connectedness on caregiver burden and depression in cancer caregivers.

## 5. Conclusions

In summary, this study provides insights into the moderating role of social connectedness on the relationship between caregiver burden and depression symptoms in caregivers of people with cancer. Caregivers will likely benefit from strategies and interventions designed to enhance perceptions of social connectedness, which in turn, have the potential to reduce caregiver burden and depression symptoms. The findings provide support for efforts and resources designed to improve caregivers’ capacity to engage with their social support networks in ways that improve their perceived sense of connectedness with others. In terms of clinical implications and future directions, investigation into whether programs designed to enhance social connectedness are effective in minimizing depression in cancer caregiver populations is warranted. Further research into domains of social support that influence anxiety, as well as examining social connectedness in older, spousal caregivers is also recommended to test for generalizability.

## Figures and Tables

**Table 1:** Sociodemographic details of 189 participants.

Sociodemographic Characteristic	Total Sample (*n* = 189)	
	*n*	%
Gender		
Male	36	19%
Female	152	80.4%
Non-binary	1	0.5%
Education		
High school (years 7–12)	27	14.3%
Vocational	47	24.9%
University (undergraduate)	77	40.7%
University (postgraduate)	38	20.1%
Speaks English at home	188	99.5%
Marital status		
Single	43	22.8%
In a relationship	134	70.9%
Divorced	7	3.7%
Widowed	5	2.6%
The care recipient is my:		
Spouse/Partner	20	10.6%
Mother/Father	79	41.8%
Other family member	40	21.1%
Friend/Other	42	22.2%
Country residing		
UK	169	89.4%
Other	20	10.6%
Illness/Disability		
None	106	56.1%
One condition	66	34.9%
Two or more conditions	17	9%
Live with care recipient		
Yes, lived with them before diagnosis	39	20.6%
Yes, began living with them after diagnosis	26	13.8%
No	124	65.6%
Care recipient cancer diagnosis type		
Blood cancer	19	10.1%
Breast cancer	42	22.2%
Gastrointestinal	17	9.0%
Genitourinary	33	17.4%
Gynecological	16	8.5%
Lung cancer	25	13.2%
Other	37	19.6%
Care recipient currently receiving treatment		
Yes	155	82%
No	34	18%

**Table 2:** Correlations between main study variables and continuous sociodemographic and care-related characteristics.

Variable	Mean	SD	Correlation Matrix					
			Depression	Anxiety	Caregiver Burden	Social Connectedness	Age	Illness/Disability
Main Study Variables								
Depression	8.99	3.29						
Anxiety	11.40	4.00	0.577 **					
Caregiver burden	15.47	5.44	0.612 **	0.522 **				
Social connectedness	26.04	7.66	−0.575 **	−0.547 **	−0.561 **			
Demographic characteristics								
Age	36.19	11.78	0.063	−0.155 *	0.093	0.074		
Number of caregiver illness/disability	0.55	0.74	0.193 **	0.163 **	0.143 **	−0.086	0.061	
Caregiver characteristics								
Caregiving duration (years)	2.44	3.37	0.129	−0.006	0.080	−0.075	0.124	0.120

* < 0.05; ** < 0.001; SD = Standard deviation.

**Table 3:** Descriptive statistics and *t*-test findings comparing residency status on main study variables.

Main Study Variable	Live with Care Recipient (*n* = 65)		Does Not Live with the Care Recipient (*n* = 124)				
	M	SD	M	SD	df	T	sig
Depression	9.71	3.59	8.62	3.09	187	−2.18	0.03
Anxiety	12.34	3.72	10.90	4.07	187	−2.37	0.02
Caregiver burden	16.88	5.85	14.73	5.09	187	−2.62	0.009
Social connectedness	23.95	7.69	27.15	7.44	187	2.77	0.006

**Table 4:** Results from the test of the moderation model of social connectedness.

Effect *, Variable	β	se	t	*p*-Value	LLCI	ULCI
Direct effect of predictor (caregiver burden) on depression	0.418	0.094	4.464	<0.001	0.233	0.603
Direct effect of moderator (perceived social connectedness) on depression	−0.041	0.059	−0.695	0.488	−0.158	0.076
Direct interaction effect (caregiver burden x perceived social connectedness) on depression	−0.007	0.003	−2.026	0.04	−0.014	−0.0002
R^2^ = 0.478, F(6, 182) = 27.747, *p* < 0.001						

* Controlling for age, caregiver illness/disability, relationship to the care recipient, residential status, care recipient receiving treatment. SE = Standard error; LLCI = Lower level confidence interval; ULCI = Upper level confidence interval.

## Data Availability

Data are stored at La Trobe University and are available on request from the first author.
